# Preparation of Absorption-Resistant Hard Tissue Using Dental Pulp-Derived Cells and Honeycomb Tricalcium Phosphate

**DOI:** 10.3390/ma14123409

**Published:** 2021-06-20

**Authors:** Kiyofumi Takabatake, Keisuke Nakano, Hotaka Kawai, Yasunori Inada, Shintaro Sukegawa, Shan Qiusheng, Shigeko Fushimi, Hidetsugu Tsujigiwa, Hitoshi Nagatsuka

**Affiliations:** 1 Department of Oral Pathology and Medicine, Graduate School of Medicine, Dentistry and Pharmaceutical Science, Okayama University, Okayama 700-8525, Japan; gmd422094@s.okayama-u.ac.jp (K.T.); de18018@s.okayama-u.ac.jp (H.K.); inayasu@s.okayama-u.ac.jp (Y.I.); gouwan19@gmail.com (S.S.); hrbmushanqiusheng@163.com (S.Q.); fushimi@med.kawasaki-m.ac.jp (S.F.); tsuji@dls.ous.ac.jp (H.T.); jin@okayama-u.ac.jp (H.N.); 2 Department of Oral and Maxillofacial Surgery, Kagawa Prefectural Central Hospital, Kagawa 760-8557, Japan; 3 Department of Life Science, Faculty of Science, Okayama University of Science, Okayama 700-0005, Japan

**Keywords:** dental pulp, mesenchymal stem cells, honeycomb TCP, matrix formation, dentin formation, osteodentin

## Abstract

In recent years, there has been increasing interest in the treatment of bone defects using undifferentiated mesenchymal stem cells (MSCs) in vivo. Recently, dental pulp has been proposed as a promising source of pluripotent mesenchymal stem cells (MSCs), which can be used in various clinical applications. Dentin is the hard tissue that makes up teeth, and has the same composition and strength as bone. However, unlike bone, dentin is usually not remodeled under physiological conditions. Here, we generated odontoblast-like cells from mouse dental pulp stem cells and combined them with honeycomb tricalcium phosphate (TCP) with a 300 μm hole to create bone-like tissue under the skin of mice. The bone-like hard tissue produced in this study was different from bone tissue, i.e., was not resorbed by osteoclasts and was less easily absorbed than the bone tissue. It has been suggested that hard tissue-forming cells induced from dental pulp do not have the ability to induce osteoclast differentiation. Therefore, the newly created bone-like hard tissue has high potential for absorption-resistant hard tissue repair and regeneration procedures.

## 1. Introduction

Autologous bone grafts and artificial biomaterials are currently used to treat bone defects in the head and neck area due to trauma, tumors, or surgical interventions. Various materials are also being developed for these purposes, owing to advances in regenerative medicine. Mesenchymal stem cells or induced pluripotent stem cells are often used as stem cell elements, and bone morphogenetic protein-2 (BMP-2) is widely used as a growth factor. In addition, much research has focused on the development of artificial biomaterials, and some are already widely used in clinical applications, including biocompatible ceramic biomaterials such as hydroxyapatite and tricalcium phosphate (TCP). The optimal geometry of artificial biomaterials is also thought to be important for inducing cell differentiation and tissue formation [1,2,3]. In recent years, attention has been focused on the treatment of bone defects using undifferentiated mesenchymal stem cells in vivo. Among mesenchymal stem cells, bone marrow-derived stem cells are considered to be the main source of stem cells, and their pluripotency makes them particularly attractive as donor cells for regenerative medicine. In vivo, the stem cells derived from bone marrow have been reported to differentiate into various cell lineages, such as intestinal mucosal epithelium, tracheal epithelium, salivary glands and brain neurons in normal tissues [4,5]. Recent studies have shown the presence of bone marrow-derived adherent cells that can be cultured for significant durations [6,7,8], which differentiate into osteoblasts, chondrocytes, and adipocytes [9,10]. As mentioned above, these bone marrow-derived cells have been applied to tissue regeneration, especially bone tissue regeneration [11,12]. Many bone marrow-derived cells are also present in the dental pulp, and dental pulp stem cells have been confirmed to also differentiate into hard tissue-forming cells. These cells also have the potential for use in bone tissue repair [13].

Bones in the maxillofacial region have a low regenerative capacity, so it is difficult for young people to completely heal when a bone defect occurs due to trauma or craniotomy. Autologous bone grafting is the most common method used in the clinic because reconstruction of the skull and jawbone requires the use of materials that have sufficient strength and biocompatibility. However, autologous bone grafts are highly invasive and can burden the patient [14,15]. Bone resorption is a major problem in mandibular regeneration and repair. Bone tissues lose their volume with age. The alveolar bone undergoes rapid reabsorption, even after artificial bone regeneration or transplantation, making it difficult to maintain sufficient bone mass. Recently, nonresorbable artificial bones, such as hydroxyapatite, have been used to repair defects in the maxillofacial region. However, nonresorbable artificial bone blocks the interaction and blood flow between the artificial bone and adjacent tissue, causing fragility, distortion of the surrounding tissue, and increased susceptibility to infection [16,17,18]. Highly biocompatible absorbent ceramics such as TCP have been shown to be effective in inducing and reconstructing bone tissue, and have been widely used in clinical applications [18,19,20,21,22,23]. However, although it is possible to induce bone tissue rapidly, it is not possible to suppress the absorption of hard tissue. Recently, dental pulp has been proposed as a promising source of pluripotent mesenchymal stem cells (MSCs), which are used in various clinical applications. So far, we have succeeded in establishing rat pulp-derived cells that can differentiate odontoblasts under in vitro conditions.

Dentin is a hard tissue that makes up teeth and has the same composition and strength as bone. However, unlike bone, dentin is usually not remodeled under physiological conditions. Therefore, it is classified as a hard tissue that is not absorbed by osteoclasts. If dentin is applied to repair a defect in the jawbone, it is expected to function as a hard tissue that is difficult to absorb. In this study, bone-like dentin was produced using stem cells collected from the pulp, and its absorption resistance was compared with that of regenerated bone tissue. This study is a preliminary report on the creation of regenerated bone that is difficult to absorb.

## 2. Materials and Methods

### 2.1. Experimental Animals

All animal experiments were approved by the Animal Experiment Control Committee of Okayama University (OKU-2011048). Twelve 4-week-old male severe combined immunodeficient (SCID) mice (CLEA Japan, Inc., Tokyo, Japan) and six-week-old GFP transgenic female SD-Tg (CAG-EGFP) mice (SHIMIZU Laboratory Supplies, Co., Ltd., Kyoto, Japan) were used in this study.

### 2.2. Honeycomb TCP Scaffolds Preparation Method

**Figure 1:**
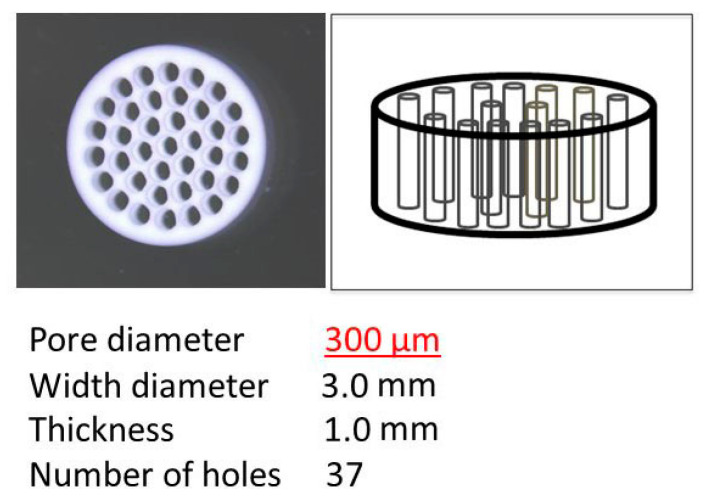
The honeycomb TCP used in the transplant experiment.

The honeycomb TCP scaffolds used in this experiment were pressed into a cylindrical shape through holes with diameters of 300 μm (300TCP), as shown in Figure 1. The details of the manufacturing method of honeycomb TCP scaffolds have been described previously [24].

### 2.3. Cell Isolation and Culture

Pulp extracted from the GFP-transgenic mice mandibular incisors was digested with a solution of 1 mg/mL dispase (Invitrogen Co., New York, NY, USA) and 1 mg/mL collagenase type II (Invitrogen Co., New York, NY, USA) for 2 h at 37 °C. The pulp cell suspensions were cultured and maintained in Dulbecco’s minimal essential medium (DMEM; Invitrogen, Carlsbad, CA, USA) supplemented with 10% Fetal Bovines Serum and 100 U/mL antimycotic-antibiotic (Life Technologies, Thermo Fisher Scientific Inc., Tokyo, Japan). The cell culture medium changed every 3 days.

Osteogenic medium was prepared by adding glycerol 2-phosphate disodium salt hydrate (β-GP) (6 mM; Sigma-Aldrich) and ascorbic acid (50 μg/mL; Sigma-Aldrich, St. Louis, MO, USA) to the standard medium. Osteogenic medium was changed every 3 days.

### 2.4. Implantation Procedure and Histological Examination

SCID mice were intraperitoneally anesthetized using dormitol (0.5 mg/kg body weight) (Meiji Co., Ltd., Tokyo, Japan) and ketamine (75 mg/kg body weight) (Fuji Chemical Industry Co., Ltd., Tokyo, Japan). Pulp-derived cells (1 × 10^7^) were cultured in osteogenic medium for 24 h, seeded onto the honeycomb TCP, and ectopically implanted into mice subcutaneously. As a control, honeycomb TCP added to BMP-2 (80 μg/mL) was implanted ectopically (subcutaneous back). The numbers of animals and samples used in the experiments are shown in Table 1. We previously conducted bone induction experiments using TCP scaffolds and reported that bone remodeling was activated 4 weeks after transplantation [25]. Therefore, in the present study, we examined the tissue samples at 4 weeks after transplantation. At 4 weeks, the experimental animals were euthanized with an isoflurane overdose and the tissues surrounding the implant sites were excised. All samples were fixed with 4% paraformaldehyde and decalcified in 10% ethylenediaminetetraacetic acid for 2 weeks. Then, the samples were embedded in paraffin and sectioned at 5 μm thickness. Finally, sections were stained with hematoxylin-eosin (H & E).

### 2.5. Immunohistochemical Staining of RANKL, CD68 and DSP

In this study, the primary antibodies goat monoclonal anti-RANKL (Santa Cruz Biotechnology, Inc., Dallas, TX, USA), rabbit polyclonal anti-CD68 (Santa Cruz Biotechnology, Inc., Dallas, TX, USA) and rabbit polyclonal anti-DSP (Santa Cruz Biotechnology, Inc., Dallas, TX, USA) were used. Immunohistochemical staining was performed as previously described [13,26]. Following antigen retrieval, the sections were incubated with normal serum for 30 min, and treated with 0.1% trypsin (Difco Laboratories, Detroit, MI, USA) for 5 min at 37 °C. Immunohistochemistry was performed using anti-RANKL (dilution; 1:200), anti-CD68 (dilution; 1:100) or anti-DSP (dilution; 1:100) for 120 min at room temperature. Tagging of primary antibodies was achieved by the subsequent application of anti-goat secondary antibody by the use of the Histofine SAB-Po^®^ Kit (Nichirei, Tokyo, Japan). Immunoreactivity was visualized using diaminobenzidine (DAB)/H_2_O_2_ solution (Histofine DAB substrate; Nichirei), and the slides were counterstained with Mayer’s hematoxylin.

### 2.6. Histological Evaluation of Hard Tissue Specimens

In the specimens prepared from each sample, the central part of the implant body was used as the observation site. The area of the formed hard tissue and the number of multinucleated giant cells (MGC) were counted using HE-stained specimens. The number of RANKL- and CD68-positive cells was counted using immunostaining specimens. Tissue observations and measurements were performed using a microscope with an image measurement software (BZ-X700, Keyence, Osaka, Japan). For morphological measurements, five arbitrary locations were selected for each sample in a high-magnification field of view and used for analysis as image data.

### 2.7. Statistics Analysis

Statistical data are shown as the mean ±SEM. Comparisons between the mean variables of the two groups were conducted by Student’s *t*-test. *p* < 0.05 was considered as statistically significant.

## 3. Results

### 3.1. Histology of Formed Hard Tissue

**Figure 2:**
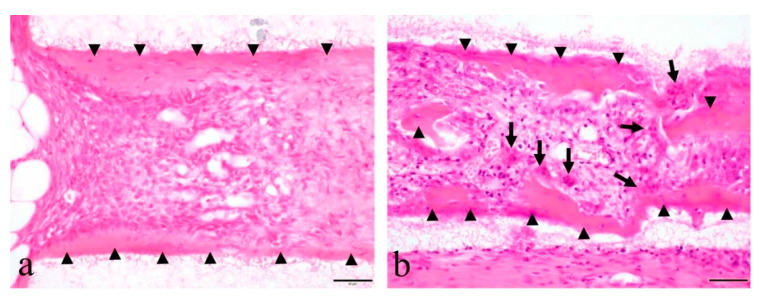
Histological images of formed hard tissues. (**a**) Hard tissue in the experimental group. (**b**) Hard tissue in the control group. Arrowhead: Bone-like hard tissue, Arrow: Multinucleated giant cell. The scale bar indicates 50 µm.

In the tissues from the experimental group, the formation of bone-like hard tissue was observed in the honeycomb TCP scaffold. An acidophilic hard tissue substrate was formed on the TCP surface. A layered, bone-like hard tissue formed with a few surface undulations. A large number of encapsulated cells were present inside the hard tissue, and cuboidal cells were densely arranged on its surface. Histologically, it had a fibrous bone shape, and no multinucleated giant cells appeared around it (Figure 2a). In the tissues of the control group, the formation of bone-like hard tissue was also observed in the honeycomb TCP scaffold. An acidophilic hard tissue substrate was continuously formed on the surface of the TCP. The formed hard tissue substrate had a large number of encapsulated cells in the substrate, and the surface was covered with cuboidal cells. The morphology of the formed hard tissue had irregular undulations, and osteoclast-like multinucleated giant cells appeared on the surface and in the surrounding areas (Figure 2b).

### 3.2. Immunohistochemistry of Formed Hard Tissue

**Figure 3:**
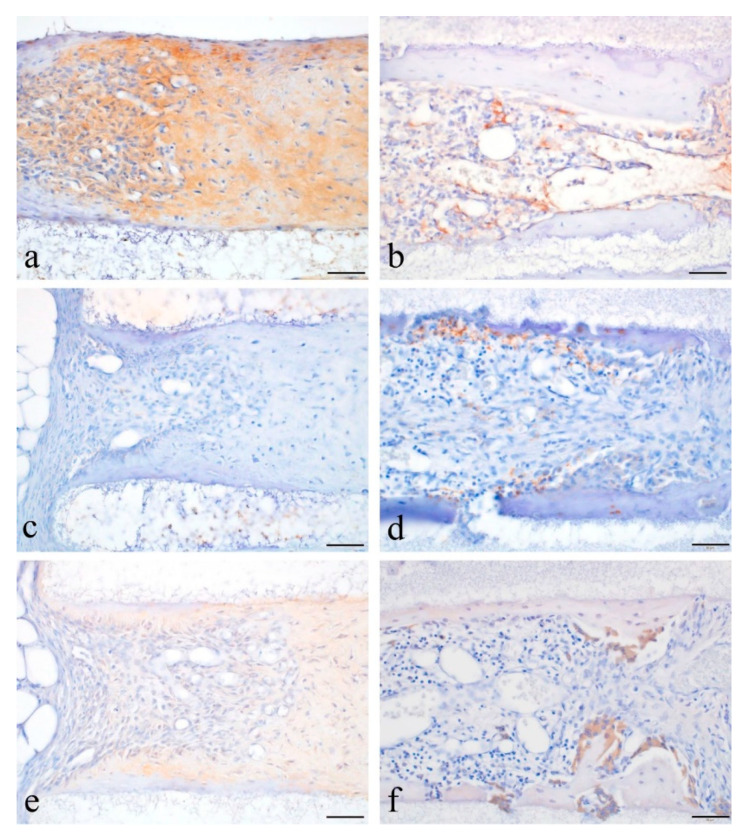
IHC images of formed hard tissues. (**a**) DSP localization in the tissues of the experimental group. (**b**) DSP localization in the tissues of the control group. (**c**) RANKL localization in the tissues of the experimental group. (**d**) RANKL localization in the tissues of the control group. (**e**) CD68 localization in the tissues of the experimental group. (**f**) CD68 localization in the tissues of the control group. The scale bar indicates 50 µm.

In the experimental group, DSP-positive reactions were observed in the formed hard tissue and in the cells inside and around the hard tissue (Figure 3a). In the control group, there were no DSP-positive reactions in the hard tissues and cells (Figure 3b). In the experimental group, there were no RANKL-positive reactions in the cells distributed around the hard tissue (Figure 3c). In the control group, cells inside and around the hard tissue had a RANKL-positive reaction (Figure 3d). In the experimental group, there were no CD68-positive reactions in the cells surrounding the hard tissue (Figure 3e). In the control group, CD68-positive reactions were observed in the multinucleated cells and in the large round-shaped cells around the hard tissue (Figure 3f).

### 3.3. Measurement of IHC-Positive Cells, Multinucleated Giant Cells and Area of Formed Hard Tissue

**Figure 4:**
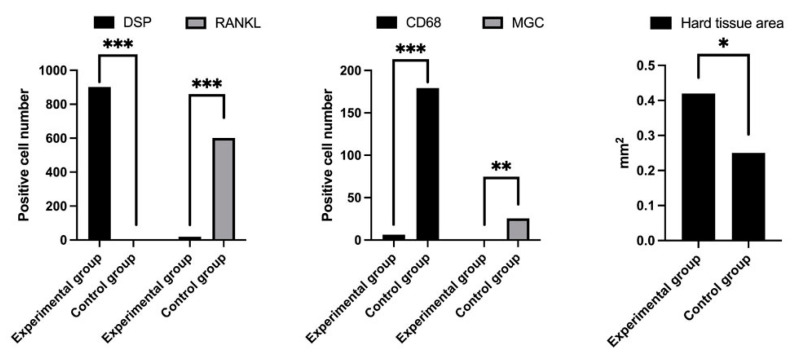
Quantification of the number of IHC-positive cells, number of MGCs, and hard tissue area in the tissue specimens. *** *p* < 0.0001, ** *p* < 0.001, * *p* < 0.05.

The amount of IHC-positive cells and hard tissue formed per unit area of the tissue samples was evaluated. The number of DSP-positive cells (experimental group 902.4, control group 0.0) was significantly higher in the experimental group (*p* < 0.0001) (Figure 4). The number of RANKL-positive cells was 19.2 and 601.6 in the experimental and control groups, respectively (Figure 4), which was significantly different (*p* < 0.0001). The number of CD68-positive cells (6.4 in the experimental group and 179.2 in the control group) was significantly higher in the control (*p* < 0.0001) (Figure 4). The number of multinucleated giant cells (0.0 in the experimental group and 25.6 in the control group) was also significantly higher in the control group (*p* < 0.01) (Figure 4). The hard tissue area (0.42 mm^2^ in the experimental group and 0.25 mm^2^ in the control group) was significantly larger in the experimental group (*p* < 0.05) (Figure 4).

## 4. Discussion

In this study, cells with hard tissue-forming ability were isolated from dental pulp stem cells and transplanted subcutaneously into mice in combination with 300TCP to form special hard tissues. Previous studies have shown that cells collected from pulp tissue differentiate into odontoblast-like cells [13]. Although many studies have been conducted on the regeneration of dentin, none has applied dentin-like hard tissue to the regeneration and restoration of bone tissue [27,28,29]. This is the first research report showing the usefulness of applying dentin-like hard tissue to bone tissue regeneration. In this study, we conducted a transplantation experiment according to the above-mentioned isolation and culture conditions for dental pulp cells. The hard tissue formed under the skin of the mouse in the experimental group had a homogeneous acidophilic substrate with no lamellar structure and a large number of encapsulated cells inside it. Usually, dentin contains dentin tubules and characteristic odontoblasts. A histological examination revealed that the hard tissue in the experimental group had no capillary structure or odontoblast-like cells, and had poor morphological characteristics similar to dentin. The hard tissue formed in the experimental group and the bone tissue formed in the control group had similar structures, but the structural difference between the two was not clear. The morphologies of the hard tissue-forming cells in the experimental group tissue were cubic or polygonal, and were morphologically similar to the osteoblasts and bone cells in the bone tissue of the control group. The hard tissue-forming cells in the tissues in the experimental group were distributed inside and on the surface of the hard tissue, and were similar to the distribution of cells observed in the bone tissue of the control group. The structure of hard tissue and the morphology and distribution of hard tissue-forming cells were not significantly different between the two groups, but their immunohistochemical profiles were significantly different. DSP was strongly expressed in the hard tissues that formed in the experimental group. DSP is a matrix protein specifically present in odontoblasts and in the dentin matrix [30], and the hard tissue formed in the experimental group was considered to have components similar to dentin. It has been reported that RANKL is expressed on the cell surface of osteocytes and osteoblasts [31]. The cells present in the hard tissue of the experimental group did not express RANKL. The hard tissue-forming cells induced in the experimental group comprised cells that were significantly different from osteoblasts, and were considered to have the properties of odontoblasts. A large number of CD68-positive cells and osteoclast-like multinucleated giant cells appeared around the bone tissue formed in the control group. In the experimental group, the number of CD68-positive cells that appeared around the hard tissue was small, and no osteoclast-like cells were found. Osteocytes and osteoblasts stimulate osteoclast progenitor cells via RANKL to induce osteoclasts [32]. Unlike osteoblasts, the cells present in the hard tissue of the experimental group were considered to be non-RANKL-expressing, and did not induce osteoclast differentiation. The hard tissue formed in the experimental group had a smooth surface with no irregularities, and there was no evidence of bone resorption by osteoclasts. The surface structure of the bone tissue formed in the control group was irregular and uneven, indicating marked bone resorption. The amount of hard tissue formed in the experimental group was smaller than the amount of bone tissue formed in the control group; therefore, it was considered that the absorption of hard tissue by osteoclasts did not occur in the experimental group. In recent years, various biomaterials with superior physical characteristics have been reported, including bioactive glass-crystalline materials and titanium mesh, etc. [33]. These materials also have potential as scaffolds for differentiating odontoblasts from dental pulp cells.

## 5. Conclusions

In this study, we were able to induce cells with the characteristics of odontoblasts from dental pulp cells to form bone-like hard tissue. This bone-like hard tissue was not resorbed by osteoclasts, and was less easily resorbed than bone tissue. It has been suggested that hard tissue-forming cells induced from the dental pulp do not have the ability to induce osteoclast differentiation. The newly created bone-like hard tissue has high potential for use in procedures for absorption-resistant hard tissue repair and regeneration.

## Figures and Tables

**Table 1:** The number of animals and samples.

Group	Number of Animals	Number of Implants
Experimental	20	40 ^1^
Control	20	40 ^1^

^1^ Implants were made in two places on the back of one individual.

## Data Availability

Not applicable.
